# Rare Primitive Lung Adenocarcinoma in Larynx: A Case Report

**DOI:** 10.1007/s12070-024-04993-1

**Published:** 2024-08-24

**Authors:** Maria Grazia Maglione, Giovanni Salzano, Francesco Maffia, Raffaele Spinelli, Emanuele Carraturo, Rossella De Cecio, Franco Ionna

**Affiliations:** 1 Department of Otolaryngology and Maxillo-Facial Surgery Unit, Istituto Nazionale Tumori—IRCCS Fondazione G. Pascale, Naples, 80131 Italy; 2 Department of Maxillofacial Surgery, Federico II University of Naples, Via Pansini, 5, Naples, 80131 Italy; 3 Department of Pathology, Istituto Nazionale Tumori—IRCCS Fondazione G. Pascale, Naples, 80131 Italy

**Keywords:** Laryngectomy, Laryngology, Oncology, Adenocarcinoma, Head and Neck Surgery

## Abstract

Laryngeal metastasis from a primitive distant cancer is a rare finding in clinical practice. In this paper is presented the case of a 63-year-old patient treated for lung adenocarcinoma metastasis in the supraglottic larynx. A review of literature suggests how the treatment of this kind of case is not unique.

## Introduction

Laryngeal metastasis from a primitive distant cancer is a rare finding in daily clinical practice [1]. Secondary laryngeal tumors from cutaneous melanoma, followed by renal cell carcinoma, appear to be most common, as Literature suggests [2, 3]. Lung metastasis to the larynx on the other hand seems to be a more than unique finding, especially when it comes from lung adenocarcinoma [3]. Furthermore, their management and treatment represent a big challenge for surgeons and physicians in general, even more, if they come as the first manifestation of primary localization [4–6]. Moreover, the anatomical position and histopathological pattern determine a great tendency to misdiagnosis, complicating the management [7, 8]. The surgical treatment of these rarities represents a challenge for the physician, due to the lack of precise guidelines [8–10]. In this paper is presented the clinical case of a 63-year-old patient treated at the Otolaryngology-Maxillofacial Surgery Department from IRCCS “Giovanni Pascale” for a primitive lung adenocarcinoma found in the supraglottic larynx. A brief review of the literature on similar cases was performed to better describe the treatment of these findings.

## Case Report

### Preoperative Findings

**Fig. 1:**
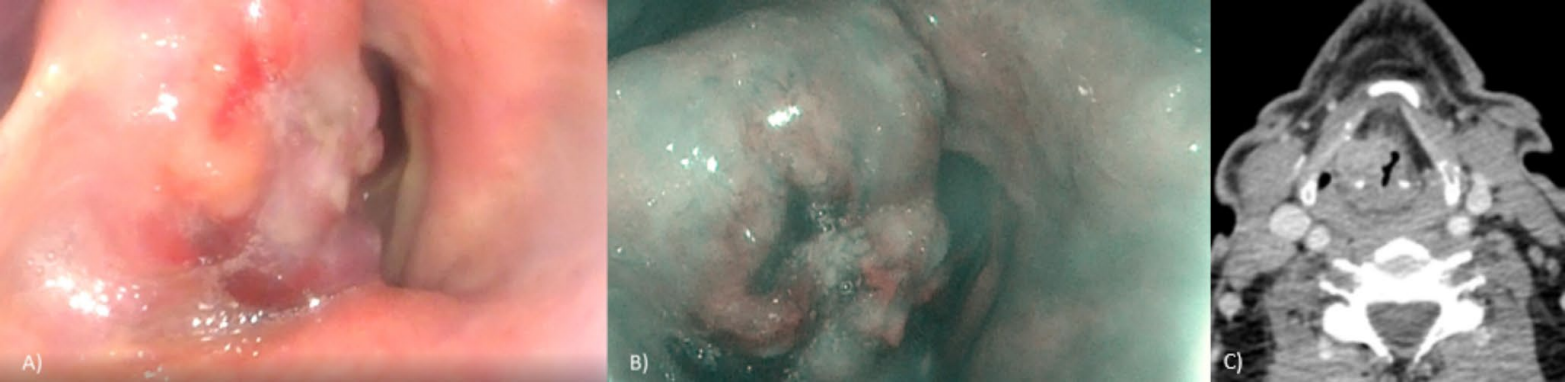
Videolaryngoscopy of the lesion in right hemilarynx (**A**); Narrow Band Imaging (NBI) of the lesion (**B**) CT scan of the lesion (axial projection) (**C**)

In March 2022, a 63 year old patient accessed the Otolaryngology-Maxillofacial Surgery Department of the IRCCS “Fondazione G. Pascale” in Naples, for dysphonia, dyspnea, solid dysphagia, and recurrent sore throat. At first examination, a mass occupying the supraglottic space was noticed through video fibrolaryngoscopy (Fig. 1, A, B).

This mass interested the whole right hemilarynx originating from the false vocal fold to the laryngeal ventricle. It appeared exophytic, dyschromic, and ulcerative and covered almost all the true vocal fold. The clinical objectivity of neck lymph nodes showed some tumefactions in the right laterocervical region. These masses appeared of solid consistency, painful, of about 2 cm in diameter. Ultrasound examination described this lesion as 24 × 12 mm in length. Computer Tomography (CT) imaging of the neck evidenced a 17 × 12 mm lesion in the right larynx, and a 22 × 14 mm coarse mass in the right laterocervical region, both positive for contrastographic enhancement (Fig. 1, C).

The patient underwent Positron Emission Tomography (PET) that evidenced radiopharmaceutical hyperaccumulation in the right laryngeal mass (Standardized Uptake Value-SUV max of 5.7) and the right laterocervical mass (SUV max of 3.6).

### Surgical Procedures

On 28th March patient underwent multiple laryngeal biopsies in CO2 laser microlaryngeal surgery. Histopathological examination diagnosed poorly differentiated adenocarcinoma. Right after this primary histopathological report patient underwent horizontal supraglottic laryngectomy along right elective neck dissection (Lv II-IV, 24 nodes neck dissection) and left elective neck dissection (lv II-IV, 26 nodes).

### Histopathology

**Fig. 2:**
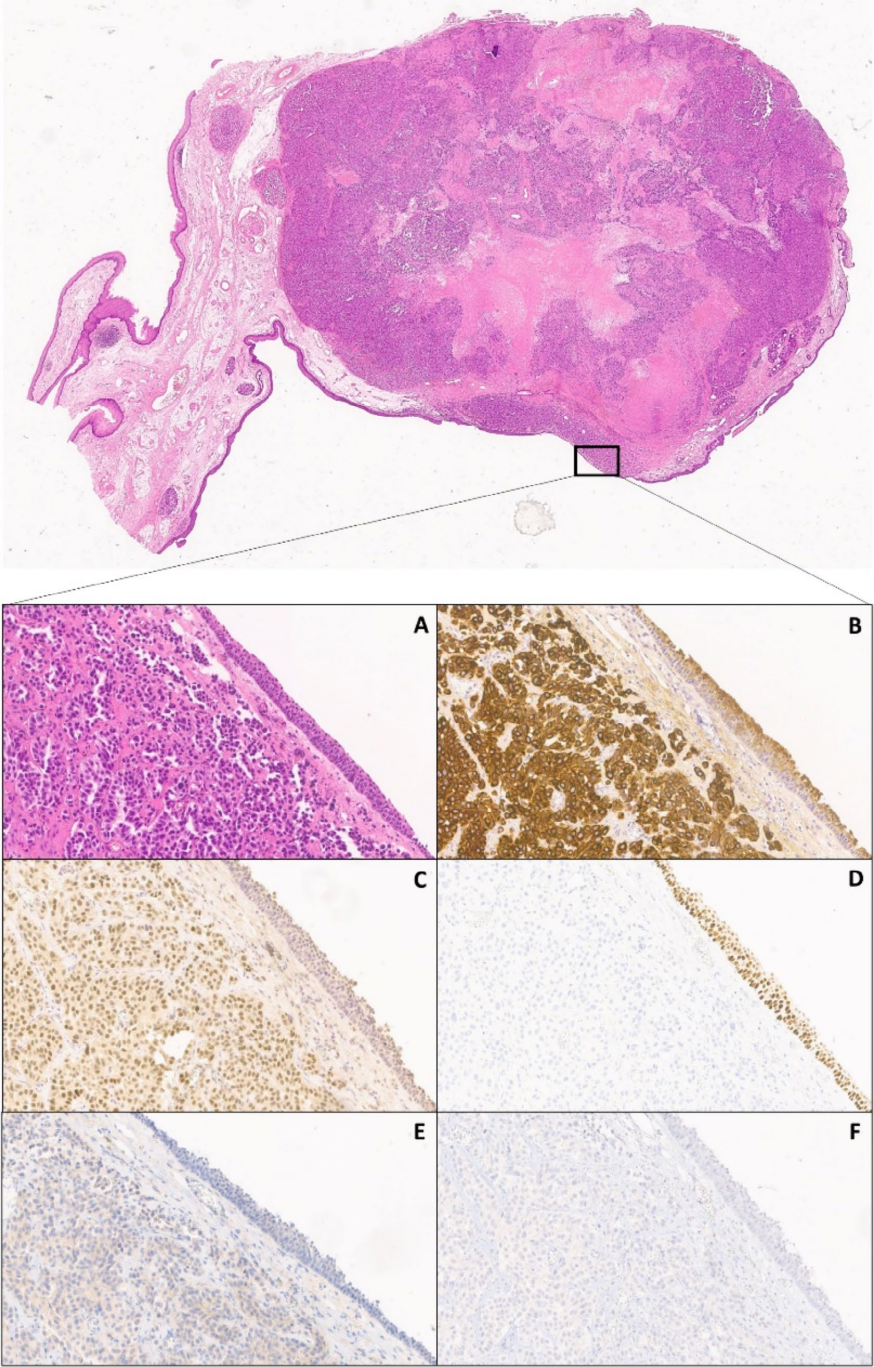
(**A**) Hematoxylin-eosin, 20X; (**B**) CK8/18, 20X; (**C**) TTF1, 20X; (**D**) P40, 20X; (**E**) PD-L1 SP263, 20X; (**F**) ALK, D5F3

Of the 24 isolated nodes from the right neck dissection, 5 (all coming from the I level) showed carcinomatous metastasis while of the 26 nodes coming from the left neck dissection, none showed ant infiltration. Nodal parenchyma (in metastatic right nodes) homed neoplasm with an acinar and trabecular pattern, positive for TTF1 and CK8-18, negative for CK19, thyroglobulin and PAX8. The surgical specimen obtained from the supraglottic laryngectomy (5 × 5 × 2 cm) showed a 2 × 1, white-gray, infiltrating lesion adjacent to the root of the right epiglottis. The final Histopatologycal report diagnosed primitive lung adenocarcinoma. A 4 × 1 × 0,5 cm fragment from the thyroid cartilage showed stromal and cartilaginous infiltration from primitive lung adenocarcinoma (TTF1 +, p40 -) (Fig. 2).

### Follow Up

After dismission, the patient was discharged with permanent tracheostomy and started speech therapy protocol for rehabilitation. The patient underwent a 6-months PET-CT scan negative for any sign of pathology resumption. Radioterapy was retained not necessary. Patient was proposed between adiuvant chemotherapy vs. strict follow-up. Educed about the complicancies, patient choosed to pursue the latter path.

## Discussion

Secondarism to the larynx represents a very rare finding, and metastasis from lung adenocarcinoma is an even rarer event [1, 2]. International Literature describes malignant melanoma as the main secondarism affecting the larynx directly followed by renal cell carcinoma. Being an almost fully cartilaginous and muscular anatomical structure justifies the rarity of metastatic spread to the larynx from distant organs. Due to his cephalic position larynx benefits from the terminal vascular and lymphatic supply [5]. Ferlito et al. (1993) described 134 cases of secondarsim to the larynx, appointing only 9 patients suffering from bronchogenic or lung metastasis (12,06%) [4]. After 1993 only 4 other cases of bronchogenic spread to the larynx have been described, showing the rarity of this particular biological spread. The brief review of literature provided here has the goal to present 4 cases of bronchogenic and lung malignancies that affected the larynx. Exclusion criteria were inherent to the language of the manuscript, as we accounted for only publications in English.

The first case (Kalai et al., 2015) described a 49 years old male patient suffering from shortness of breath and loss of weight, diagnosed with a lung tumor that showed a small nodule on the inferior surface of the epiglottis. Despite the Histopathological diagnosis of squamous cell carcinoma, the laryngeal metastasis, being asymptomatic, was not directly treated. The treatment was based on platinum‑based doublet chemotherapy (Paclitaxel with Cisplatin) [5].

Excluded this particular case, the other three publications included in this review share some typical aspects, both from the clinical and surgical point of view: Bernaldez et al. (1994) described a 56 years old male with a known history of epidermoid carcinoma of the left lung underwent total laryngectomy with left radical neck dissection and right functional neck dissection for a 2 × 2 cm polypoid lesion in the left aryepiglottic fold [1].

Nicolai et al. (1996) described a 69-year-old patient with dysphonia due to a transglottic mass with partial destruction of the cricoid cartilage. At the same time, an additional lesion in the apical lobe of the right lung was discovered. Histopathological examination of both lesions showed poorly differentiated adenocarcinoma. The patient finally underwent a total laryngectomy extended to the first two tracheal rings, bilateral functional neck dissection, and total thyroidectomy after the failure of radiotherapy [3].

Ogata et al. (1993) described a 59-year female with a history of adenocarcinoma of the lower lung treated with lobectomy. The patient suffered from a 5 mm hemangioma-like lesion with a smooth surface beneath the anterior commissure of the larynx. Histopathological examination confirmed the diagnosis of adenocarcinoma and she finally underwent a partial laryngectomy [6] (Table 1).

**Table 1:** Brief review of literature about bronchogenic secondarisms to the larynx

Title and Authorship	Year of Publication	Laryngeal Symptoms	Treatment	Definitive Histopathological Diagnosis
*Ogata H*, et al. Laryngeal metastasis from a pulmonary papillary adenocarcinoma: a case report.	1993	Hoarseness, Breathly voice	Partial laryngectomy (supraglottic resection)	Adenocarcinoma
*Bernáldez R* et *al*, Pulmonary carcinoma metastatic to the larynx.	1994	Dysphagia	Total laryngectomy with left radical neck dissection and right functional neck dissection	Epidermoid Carcinoma
*Nicolai P* et *al*. Metastatic neoplasms to the larynx: report of three cases.	1996	Dysphonia	Total laryngectomy, bilateral functional neck dissection, and total thyroidectomy;	Adenocarcinoma
*Kalai U* et *al*, Laryngeal metastasis from lung cancer	2015	None	No specific intervention was performed for the laryngeal nodule.	Squamous Cell Carcinoma

Of the 4 case reports, only Kalai et al. did not describe a patient whose main symptom was related to phonation. This was the only patient in the review who did not benefit from any surgical treatment, as the laryngeal metastasis was asymptomatic. This example carries the first possible indication to justify the surgical treatment of this kind of lesion, that is the clinical behavior of the mass. International literature agrees on another fundamental parameter to justify partial or total laryngectomy, whereas the tumor represents a single metastasis and other organs had not been affected [1, 4, 6, 7] The CT and PET-CT scans of the patient presented in this.

manuscript showed no other secondarism except for the right hemilarynx and a right laterocervical mass, leading to the decision of performing at first right hemilaryngeal biopsy.

Once got the histopathological diagnosis supraglottic laryngectomy with prophylactic functional right and left neck dissection was performed. As the prognosis of this kind of patients is often unfortunate depending on the lymphatic vessels spread through the body, post-operative chemotherapy assumes an even more important role in the management of the lung secondarism to the larynx [10].

## Conclusion

Considering our experience and the review of the literature that has been provided, the therapeutical strategy of this particular kind of case is not unique, therefore the treatment must be tailored to the single patient.
